# The clinical characteristics, treatment, and survival of portopulmonary hypertension in Japan

**DOI:** 10.1186/s12890-021-01452-3

**Published:** 2021-03-16

**Authors:** Yukiko Takahashi, Keiko Yamamoto, Seiichiro Sakao, Takao Takeuchi, Rika Suda, Nobuhiro Tanabe, Koichiro Tatsumi

**Affiliations:** 1 Department of Respirology, Graduate School of Medicine, Chiba University, 1-8-1 Inohana, Chuou-ku, Chiba, Chiba 260-8670 Japan; 2 Department of Respirology, Saiseikai Narashino Hospital, Narashino, Japan; 3 Department of Respirology, Nissan Tamagawa Hospital, Tokyo, Japan

**Keywords:** Portopulmonary hypertension, Portal hypertension, Pulmonary arterial hypertension, PoPH

## Abstract

**Background:**

Portopulmonary hypertension (PoPH) refers to the simultaneous presentation of pulmonary arterial and portal hypertension. However, few reports have included the characteristics and treatments for patients with PoPH of Asian population; thus, we investigated the clinical characteristics, treatment, and survival of these patients in a Japanese cohort.

**Methods:**

Pulmonary arterial hypertension (PAH) has been included in the National Research Project on Intractable Disease in Japan; therefore, we extracted data of patients with PoPH from the forms of newly registered cases of the project from 2012 to 2013 (for 2 years), and updated cases of the project in 2013 (Study 1, n = 36 newly registered forms, n = 46 updated forms). Additionally, for Study 2, we performed a retrospective, observational cohort study at Chiba University Hospital (n = 11). We compared the characteristics between patients with PoPH and those with idiopathic/heritable PAH (I/H-PAH).

**Results:**

Both studies showed higher cardiac outputs (COs) and cardiac indexes (CIs), lower pulmonary vascular resistance (PVR), and less treated with combination therapy in patients with PoPH than those with I/H-PAH. In Study 2, the overall and disease-specific survival between PoPH and I/H-PAH were similar. Conversely, many patients (45%) had to change their PAH-specific medicine because of adverse effects.

**Conclusion:**

As seen in western countries, Japanese patients with PoPH showed higher COs and CIs, better exercise tolerance, and lower PVRs than patients with I/H-PAH. Further studies are needed to improve PoPH treatments.

## Introduction

Portopulmonary hypertension (PoPH) is a state of simultaneous pulmonary arterial and portal hypertension. PoPH diagnosis requires portal hypertension (not necessarily the presence of cirrhosis) and pulmonary arterial hypertension (PAH). Patients with PoPH often have high cardiac output (CO) owing to shunts and systemic vasodilation, and their initial PVR is not very high [1]. PVR gradually increases with disease progression. The severities of liver disease and those of PoPH are not necessarily correlated with each other [2, 3].

In the process, shear stress on the pulmonary vessels gradually increases, resulting in endothelial proliferation and remodeling of the pulmonary artery in PoPH. Vasoactive substance imbalances also affect pulmonary circulation owing to portosystemic shunts or defective hepatic metabolism, resulting in pathological pulmonary vascular lesions [4–6].

According to reports from western countries, PoPH accounts for 5–10% of PAH [7]. With respect to hepatic disease, PoPH accounts for 1–2% of the cases of cirrhosis, and 2–6% of the cases of portal hypertension [3, 8–10].

Previous studies have suggested that the survival of patients with PoPH was worse than that of patients with IPAH. In the REVEAL registry, 5-year survival measured from diagnosis was significantly worse in patients with PoPH than in patients with IPAH (40% vs. 64%) [11]. The Mayo clinic reported that 5-year survival was 14% for patients with PoPH not receiving PAH-targeted therapy [12]. In the United Kingdom national registry, patients with PoPH had 5-year survival rates of 35%. There was no difference in survival rates between patients with and without cirrhosis [13]. On the contrary, a French study reported that patients with cirrhosis showed better survival than patients without cirrhosis [14]. However, both studies found that the Child–Pugh Score C was associated with poor survival.

Application of PAH-targeted therapy has not been established yet for the treatment of PoPH. A recent randomized controlled study showed improved PVR at 12 weeks in the macitentan group compared to the placebo group [15]. Some studies showed that patients with PoPH tended to have initial monotherapy instead of combination therapy [14, 16]. Combination therapy was not recommended for PoPH in the 6th world symposium on Pulmonary Hypertension [17].

We aimed to investigate the clinical characteristics, treatment, and survival of patients with PoPH in a Japanese cohort because few reports of PoPH exist from Asian countries.

## Methods

### Patients

We obtained data from a nationwide registration system of patients with PAH in the first study (Study 1). In Japan, PAH was included in the National Research Project of Intractable Diseases in 2009. Patients with PAH have to submit an updated clinical research form filled out by their attending physicians every year to receive medical subsidies. Using these clinical research forms, the Respiratory Failure Research Group at the Ministry of Health and Welfare of Japan directs an epidemiological survey. We used the clinical research forms that were newly registered in 2012–2013. We analyzed 2 years’ worth of data because PoPH is a rare disease and the number of newly registered patients in a given year was small. Incident cases (included in the “registration form”) and prevalent cases that were updated in 2013 (included in the “updated form”) were included. These are the most recent ones available. Study 1 was designed as a retrospective cohort study. Pre-capillary PAH was defined as a mean pulmonary artery pressure (mPAP) of ≥ 25 mmHg, pulmonary artery wedge pressure (PAWP) of < 15 mmHg, and pulmonary vascular resistance (PVR) of ≥ 240 dyne/s/cm^−5^ [4, 18, 19]. PoPH was defined as the combination of pulmonary hypertension with portal hypertension. The diagnosis of portal hypertension was performed by the attending physician (no data on portal pressure was present in this registry). We excluded groups 3 (PH due to lung diseases and/or hypoxia) and 4 (PH due to pulmonary artery obstructions) according to the NICE classification of pulmonary hypertension [20]. The registration form contained 680 patients (36 with PoPH, 382 with idiopathic/heritable PAH [I/H-PAH], and 262 with others). The updated form contained 1071 patients (46 with PoPH, 730 with I/H-PAH, and 295 with others). We compared data of patients with PoPH and those of patients with I/H-PAH.

From the registration form, we obtained data on sex, age, hemodynamics (systolic pulmonary arterial pressure, diastolic pulmonary arterial pressure, mPAP, PVR, PAWP, right atrial pressure, CO, cardiac index [CI], and mixed venous oxygen pressure), six-minute walk distance (6MWD), blood examination results (brain natriuretic peptide [BNP] and uric acid), trans-tricuspid pressure gradient, history of right heart failure, the New York Heart Association (NYHA) functional classification, and treatment with PAH-specific drugs (modern PAH therapy) based on the PAH classification. We defined “modern PAH therapy” as treatment with endothelin-receptor antagonists (ERAs: bosentan, ambrisentan, and macitentan), phosphodiesterase type 5 inhibitors (PDE5is: sildenafil and tadalafil), soluble guanylate cyclase stimulants (i.e., riociguat), prostaglandin I2 receptor agonists (i.e., selexipag), or prostaglandin I2 analogs (i.e., epoprostenol and treprostinil). Contrary to the registration form, the updated form did not contain hemodynamics data. From the updated form, we obtained data on sex, age, 6MWD, blood examination results, trans-tricuspid pressure gradient, history of right heart failure, NYHA functional classification, and treatment with PAH-specific drugs.

The second part of our study (Study 2) was designed as a retrospective single-center cohort study at Chiba University Hospital. PAH was diagnosed based on mPAP > 20 mmHg, PVR ≥ 240 dyne/s/cm^−5^, and PAWP ≤ 15 mmHg at rest, as measured by right heart catheterization (RHC), reflecting the 2018 World Symposium guidelines [20]. PoPH was defined as the combination of pulmonary hypertension with portal hypertension (portal pressure ≥ 10 mmHg). We compared data from 11 patients with PoPH and 39 patients with I/H-PAH, who were evaluated and diagnosed between 1999 and 2017. Only one patient with IPAH met the criteria of 20 < mPAP < 25 mmHg.

We excluded patients with severe obstructive pulmonary impairment (forced expiratory volume in 1 s [FEV_1_]/forced vital capacity < 70% and FEV_1_ < 50% of predicted), or severe restrictive pulmonary impairment (vital capacity < 50% of predicted) [21] and those with clinically suspected hepatopulmonary syndrome. We also excluded group 3 and 4. We analyzed demographics, hemodynamics, blood gas analysis, 6MWD, pulmonary function, and World Health Organization functional class. For Study 2, we divided the patients into two periods based on the time of diagnosis (1999–2010 and 2011–2017) to distinguish patients with different treatment choices (epoprostenol, bosentan, sildenafil, tadalafil, and ambrisentan were approved for use in Japan in 1999, 2005, 2008, 2009, and 2010, respectively). By the end of November 2019, we collected follow-up data from 28 of 50 patients by either contacting them or their primary physicians. The remaining 22 patients were censored at the final visit date by their primary physician. The mean follow-up period was 6.6 years.

For some patients with PoPH, we could analyze the changes in variables (mPAP, PVR, CI, and medication) from baseline to the follow-up RHC.

### Statistical analysis

We used Student’s t-tests to compare continuous variables and chi-square tests to compare categorical variables and evaluate baseline differences between the two groups. We displayed results as means ± SDs or medians (interquartile ranges) for continuous variables and numbers (%) for categorical variables. We estimated survival using the Kaplan–Meier method and compared values using the log-rank test based on overall mortality and disease-specific mortality. We set the threshold for significance at p-value of < 0.05.

We performed all analyses using the JMP Pro 15.1.0, Japanese version, SAS Institute.

## Results

### Study of Baseline characteristics of Japanese patients with PoPH and I/H-PAH (Study 1)

#### Baseline characteristics of newly registered patients with PoPH or I/H-PAH based on the registration forms from 2012 to 2013 (for 2 years)

Table 1 summarizes the baseline characteristics of newly registered patients with PoPH (n = 36) and I/H-PAH (n = 382) from 2012 to 2013 (for 2 years). Patients with PoPH had lower mean ages at diagnosis, higher COs (4.7 ± 1.7 vs. 3.9 ± 1.4 L/min; *p* = 0.0086), and better 6MWD (336.5 ± 15.9 vs. 263.7 ± 145.2 m; *p* = 0.0368) than those with I/H-PAH. In the PoPH group, the ratio of females was higher than that of males (20/16 [56%]), similar to that in I/H-PAH (219/163 [57%]). We found similar mPAPs (46.1 ± 13.0 vs. 43.9 ± 13.4 mmHg; *p* = 0.3482) and treatments with combination therapy (4/32 [11%] vs. 75/307 [20%]; *p* = 0.1856) in both groups.

**Table 1:** Baseline characteristics of newly registered patients using registration forms in 2012–2013 (for 2 years) (PoPH and I/H-PAH)

	PoPH	I/H-PAH	*p* value
Number	36	382	
Sex (F/M)	20/16	219/163	0.8373
Age at diagnosis (years)	50.4 ± 17.7	58.0 ± 20.8	0.0347
Age of onset (years)	50.2 ± 20.3	55.1 ± 21.7	0.2544
Onset—First visit (months)	3.6 ± 3.4	3.4 ± 3.1	0.7624
Onset—Diagnosis (years)	2.3 ± 6.0	1.9 ± 6.7	0.6987
*Hemodynamics*			
sPAP (mmHg)	72.1 ± 19.7	69.3 ± 20.1	0.4469
dPAP (mmHg)	29.5 ± 11.1	28.6 ± 11.1	0.6790
mPAP (mmHg)	46.1 ± 13.0	43.9 ± 13.4	0.3482
PAWP (mmHg)	9.0 ± 3.1	9.8 ± 4.8	0.3321
RAP (mmHg)	8.3 ± 5.5	8.2 ± 5.5	0.9373
PVR (dyne/s/cm^−5^)	723.6 ± 483.5	830.1 ± 574.1	0.3011
CO (L/min)	4.7 ± 1.7	3.9 ± 1.4	0.0086
CI (L/min/m^2^)	2.8 ± 0.8	2.6 ± 1.0	0.2316
PvO_2_ (mmHg)	42.2 ± 7.7	42.7 ± 13.8	0.9172
PvO_2_ (%)	71.1 ± 11.6	70.5 ± 14.6	0.8313
*6-min walk test*			
6MWD (m)	336.5 ± 15.9	263.7 ± 145.2	0.0368
Lowest SpO_2_ (%)	86.7 ± 9.7	87.0 ± 7.8	0.8608
*Blood exam*			
BNP (pg/ml)	600.6 ± 950.6	487.1 ± 593.2	0.3177
UA (mg/dl)	6.3 ± 1.7	6.8 ± 2.4	0.2704
TRPG (mmHg)	66.5 ± 19.9	66.5 ± 23.1	0.9861
History of right heart failure (±)	14/20	149/229	0.8411
NYHA (1/2/3/4)	2/14/16/4	11/115/185/61	
Modern PAH therapy (±) (%)	19/17 [53]	228/154 [60]	0.4230
IV PGI_2_ (±) (%)	1/35 [3]	19/363 [5]	0.5260
ERA (±) (%)	9/27 [25]	139/243 [36]	0.1613
PDE5i (±) (%)	14/22 [39]	151/231 [40]	0.9401
Combination therapy (±) (%)	4/32 [11]	75/307 [20]	0.1856

Data provided as mean ± SD or n

sPAP, systolic pulmonary arterial pressure; dPAP, diastolic pulmonary arterial pressure; mPAP, mean pulmonary arterial pressure; PAWP, pulmonary arterial wedge pressure; RAP, right atrial pressure; PVR, pulmonary vascular resistance; CO, cardiac output; CI, cardiac index; PvO2, mixed venous oxygen pressure; 6MWD, 6-min walk distance; BNP, brain natriuretic peptide; UA, uric acid; TRPG, trans-tricuspid pressure gradient; NYHA, New York Heart Association Functional Classification; PGI_2_, prostaglandin I2; IV, intravenous; ERA, endothelin-receptor antagonist; PDE5i, phosphodiesterase type 5 inhibitor

#### Characteristics of Japanese patients with PoPH or I/H-PAH based on updated forms in 2013

Table 2 lists the baseline characteristics of patients with PoPH (n = 46) or I/H-PAH (n = 730) based on updated forms in 2013.

**Table 2:** Baseline characteristics of patients in updated forms in 2013 (PoPH and I/H-PAH)

	PoPH	I/H-PAH	*p* value
Number	46	730	
Sex (F/M)	26/20	492/238	0.1365
Age (years)	52.9 ± 17.5	52.4 ± 20.0	0.8742
Age of onset (years)	45.4 ± 20.8	44.1 ± 22.4	0.7484
*6-min walk test*			
6MWD (m)	382.8 ± 134.0	379.5 ± 136.2	0.9255
Lowest SpO_2_ (%)	92.8 ± 4.2	91.1 ± 6.6	0.2469
*Blood exam*			
BNP (pg/ml)	104.2 ± 280.8	127.0 ± 239.8	0.5590
UA (mg/dl)	6.0 ± 2.1	5.9 ± 1.9	0.7175
TRPG (mmHg)	48.8 ± 21.6	55.8 ± 24.9	0.0869
History of right heart failure (±)	19/27	397/327	0.0746
NYHA (1/2/3/4)	8/27/10/0	51/420/211/35	
Modern PAH therapy (±) (%)	41/5 [89]	615/115 [84]	0.3535
IV PGI_2_ (±) (%)	1/45 [2]	148/582 [20]	0.0003
ERA (±) (%)	24/22 [52]	503/227 [69]	0.0221
PDE5i (±) (%)	34/12 [74]	448/282 [61]	0.0811
Combination therapy (±) [%]	18/28 [39]	393/337 [54]	0.0524

Data provided as mean ± SD or n

Abbreviations are defined in Table 1

The ratio of females was higher than that of males in both the PoPH and I/H-PAH groups (26/20 [57%] vs. 492/238 [67%]). We found similar modern PAH therapy treatments in both groups, but a significant difference in terms of the combination therapy between the two groups (39% vs. 54%; *p* = 0.0524). Usage rates of PGI_2_ and ERA were lower and those of PDE5i were higher in patients with PoPH than in those with I/H-PAH.

### Study of PoPH patients at Chiba University Hospital (Study 2)

#### Baseline characteristics of patients with PoPH or I/H-PAH at diagnosis (study at Chiba University Hospital)

We analyzed variables in 11 patients with PoPH and 39 with I/H-PAH diagnosed at Chiba University Hospital between 1999 and 2017. Table 3 presents their baseline characteristics at diagnosis.

**Table 3:** Baseline characteristics of patients from the study at Chiba University Hospital (PoPH and I/H-PAH)

	PoPH	I/H-PAH	*p* value
Number	11	39	
Sex (F/M)	8/3	28/11	0.9514
Age (years)	45.8 ± 11.8	49.1 ± 18.2	0.5812
Diagnosis (1999–2010/2011–2017)	10/1	17/22	0.0029
Aetiology of PoPH (n (%))			
PoPH with cirrhosis	6 (55)		
Child–Pugh (A, B, C)	5, 0, 1		
HCV	4 (36)		
Primary biliary cirrhosis and Autoimmune	1 (9)		
Biliary atresia	1 (9)		
PoPH without cirrhosis	5 (45)		
Cryptogenic	2 (18)		
Congenital portosystemic venous shunt	3 (27)		
Haemodynamics			
mPAP (mmHg)	48.9 ± 10.5	46.5 ± 14.4	0.6065
PVR (dyne/s/cm^−5^)	772.5 ± 617.4	820.5 ± 443.7	0.7730
PAWP (mmHg)	7.2 ± 3.1	7.8 ± 3.2	0.5489
RAP (mmHg)	7.1 ± 6.0	5.4 ± 3.5	0.2348
CO (L/min)	5.29 ± 1.6	4.05 ± 0.9	0.0016
CI (L/min/m^2^)	3.1 ± 0.8	2.6 ± 0.5	0.0067
6 min walk test			
6MWD (m)	374.0 ± 84.0	413.4 ± 107.1	0.3176
Lowest SPO_2_ (%)	89.3 ± 3.7	84.5 ± 10.1	0.1791
Blood gas analysis (room air)			
PaO_2_ (mmHg)	69.2 ± 6.8	72.3 ± 16.5	0.5462
PaCO_2_ (mmHg)	34.3 ± 5.3	35.8 ± 4.4	0.3551
PvO_2_ (mmHg)	36.3 ± 5.3	35.6 ± 3.9	0.6359
Qs/Qt	22.8 ± 7.0	22.3 ± 16.1	0.9085
Pulmonary function			
VC%predicted	89.1 ± 15.0	94.4 ± 16.6	0.3661
FEV_1/_FVC%	76.9 ± 11.0	78.0 ± 8.7	0.7416
RV/TLC%	37.6 ± 8.4	36.0 ± 6.4	0.5193
DL_CO_%predicted	64.0 ± 9.3	61.1 ± 20.6	0.6741
DL_CO_/V_A_%predicted	76.0 ± 13.0	68.2 ± 22.4	0.3018
DL_CO_%predicted (adjustment for Hb)	58.2 ± 21.0	53.5 ± 28.5	0.6133
DL_CO_/V_A_%predicted (adjustment for Hb)	69.0 ± 25.2	57.7 ± 32.0	0.2839
Blood Exam			
Hemoglobin (g/dl)	13.7 ± 1.7	13.9 ± 2.1	0.8756
Creatinine (mg/dl)	0.8 ± 0.3	0.7 ± 0.3	0.8844
Total Bilirubin (mg/dl)	1.4 ± 0.8	1.0 ± 0.5	0.0450
BNP (pg/ml)	297.0 ± 485.3	170.3 ± 263.2	0.2799
Smoking habits			
Never/ Former or current (±)	4/6	14/25	0.8111
WHO FC (I/II/III/IV)	1/6/3/0	4/21/10/1	
Comorbidity			
BMI ≥ 25 kg/m^2^ (±)	3/8	9/30	0.7758
Systemic hypertension (±)	1/10	3/36	0.8816
Coronary artery disease (±)	0/11	2/37	0.3132
Atrial fibrillation (±)	2/9	2/37	0.1964
Diabetes mellitus (±)	1/10	3/36	0.8816
Number of Comorbidities			
Two (±)	1/10	5/34	0.7295
Three (±)	0/11	1/38	0.4783

Data provided as mean ± SD or n

HCV, hepatitis c virus; mPAP, mean pulmonary arterial pressure; PVR, pulmonary vascular resistance; PAWP, pulmonary arterial wedge pressure; RAP, right atrial pressure; CI, cardiac index; PvO_2_, mixed venous oxygen pressure; 6MWD, 6-min walk distance; VC, vital capacity; FEV_1_, forced expiratory volume in 1 s; RV, residual volume; TLC; total lung capacity; DLco; diffusing capacity of the lung for carbon monoxide; BNP, brain natriuretic peptide; WHO FC, World Health Organization functional class

Patients with PoPH had a high female-to-male ratio (8/3 [73%]) similar to that in patients with I/H-PAH. Regarding etiology, patients with PoPH included four with hepatitis C virus (HCV) infection, three with congenital portosystemic venous shunt, two with cryptogenic disease, one with overlap of primary biliary cirrhosis and autoimmune disease, and one with biliary atresia. Six patients (55%) had cirrhosis. We classified five patients as A and one as C according to their Child–Pugh scores. None of the patients with PoPH had received liver transplants.

The patients with PoPH had higher COs (5.3 ± 1.6 vs. 4.1 ± 0.9 L/min; p = 0.0016), CIs (3.1 ± 0.8 vs. 2.6 ± 0.5 L/min/m^2^; *p* = 0.0067), and total bilirubin values (1.4 ± 0.8 vs. 1.0 ± 0.5 mg/dl; *p* = 0.0450) than those with I/H-PAH. Patients with PoPH showed lower PVRs than those with I/H-PAH, albeit not significantly.

#### Treatment and follow-up data (study at Chiba University Hospital)

**Fig. 1:**
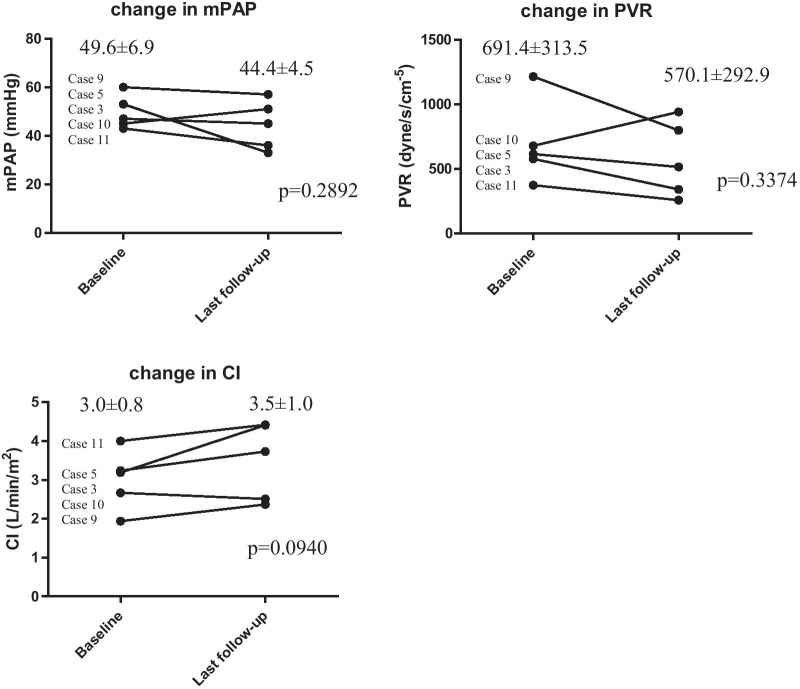
Hemodynamic Change from baseline to the last follow-up in Study 2 (study at Chiba University Hospital) (PoPH, n = 5 [case 3, 5, 9, 10 and 11]). Data provided as mean ± SD or n. Abbreviations are defined in Table 1

At their final visit, the patients with PoPH had been treated less often with combination therapy (27% vs. 67%; *p* = 0.0191) or with IV PGI_2_ and PDE5i than the patients with I/H-PAH (Table 4). Table 5 shows the medical therapies and side effects of each patient with PoPH. All patients with PoPH who received modern PAH therapies were initiated on monotherapy at the beginning of the treatment. At the final visit, 7 of the 11 patients received modern PAH therapy, and 3 of them received combination therapy. Four of them were forced to change their initial medicine to a new one owing to side effects. The most used drug was ERA, and 45% of patients with PoPH were eventually treated with it. The patient with Child–Pugh C scores did not use PAH-targeted medications. Five of the 11 patients underwent follow-up RHCs. The hemodynamic states improved in four patients (albeit insignificantly) from baseline to the last follow-up RHC (Fig. 1).

**Table 4:** PAH therapy at the time of final visit in the study at Chiba University Hospital (PoPH and I/H-PAH)

	PoPH	I/H-PAH	*p* value
Modern PAH therapy (±) (%)	7/4 [64]	33/6 [85]	0.1442
IV PGI_2_ (±) (%)	0/11 [0]	10/29 [26]	0.0176
ERA (±) (%)	5/6 [45]	22/17 [56]	0.5202
PDE5i (±) (%)	4/7 [36]	28/11 [72]	0.0335
PGI_2_ receptor agonist (±) (%)	1/10 [1]	11/28 [28]	0.1567
Combination therapy (±) (%)	3/8 [27]	26/13 [67]	0.0191

PGI_2_, prostaglandin I2; ERA, endothelin-receptor antagonist; PDE5i, phosphodiesterase type 5 inhibitor

**Table 5:** Baseline characteristics, treatment at the final visit, and outcome of patients with PoPH

Case	Etiology	Sex	Child–Pugh	mPAP (mmHg)	PVR (dyne/s/cm^−5^)	CI (L/min/m^2^)	6MWD (m)	Medical therapy (mg/day)	Discontinuation by side effects	Follow-up period (years)	Outcome
1	Cryptogenic	M	–	40	621.6	2.99		None		2.4	Died (sepsis)
2	Biliary atresia	F	A	56	563.9	3.57	525	None		15.6	
3	Extra-hepatic portal obstruction	F	–	47	577.2	3.19		Macitentan(10), Sildenafil(60), Selexipag (2.4)	Tadarafil (low blood pressure, headache), Riociguat (low blood pressure)	17.2	↓mPAP, PVR
4	HCV	F	C	46	537.5	3.59	370	None		0.3	
5	Extrahepatic portal obstruction	F	–	53	614.0	3.24	360	Ambrisentan (10)	Sildenafil (headache, dizziness), Riociguat (hepatic dysfunction), Selexipag (hepatic dysfunction, nausea, tinnitus, malaise, face redness)	11.3	↓mPAP, PVR
6	HCV	F	A	33	339.3	4.01	306	None		2	
7	HCV	F	A	44	477.0	3.86	436	Macitentan (5), Sildenafil (60)	Ambrisentan (thrombocytopenia)	10.9	
8	PBC and Autoimmune	F	A	71	2501.4	1.48	267	Sildenafil (60)		0.2	Died (heart failure)
9	HCV	M	A	60	1213.8	1.94	430	Riociguat (6)	Tadarafil (hepatic dysfunction)	14.7	↓mPAP, PVR
10	Cryptogenic	F	–	45	678.3	2.67	275	Bosentan (125), Sildenafil (60)		5.6	↑mPAP, PVR Died (heart failure)
11	Extrahepatic portal obstruction	M	–	43	373.5	4.00	397	Macitentan (10)		3.7	↓mPAP, PVR

Abbreviations are defined in Table 1. PBC: primary biliary cirrhosis

#### Survival (study at Chiba University Hospital)

**Fig. 2:**
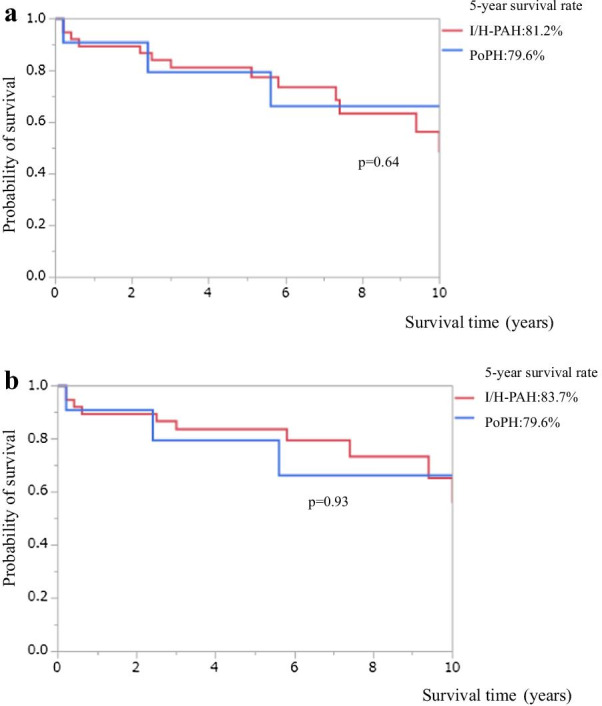
Survival in Study 2 (study at Chiba University Hospital). **a** Overall survival in PoPH and I/H-PAH (PoPH; n = 11, I/H-PAH: n = 39). There was no significant difference in overall survival between patients with PoPH and patients with I/H-PAH (5-year survival: 79.6% vs. 81.2%, p = 0.64). **b** Disease-specific survival in PoPH and I/H-PAH (PoPH; n = 11, I/H-PAH; n = 39). There was no significant difference in disease-specific survival between patients with PoPH and patients with I/H-PAH (5-year survival: 79.6% vs. 83.7%, *p* = 0.93)

Among 50 patients with PoPH or I/H-PAH, 13 patients died from PAH-related causes and 3 died from other causes during the follow ups. Regarding the patients with PoPH, 3 died (two from right heart failure and one from sepsis). We found similar overall survival between PoPH and I/H-PAH groups (5-year survivals, 79.6% vs. 81.2%, respectively; *p* = 0.64) (Fig. 2a). We also found similar disease-specific survivals between PoPH and I/H-PAH groups (5-year survivals, 79.6% vs. 83.7%, respectively; *p* = 0.93) (Fig. 2-b).

## Discussion

We conducted the largest study to date on Japanese patients with PoPH to reveal their characteristics trends. We demonstrated that patients with PoPH tended to receive monotherapy rather than combination therapy. However, the survival of patients with PoPH showed no significant difference compared to that of patients with I/H-PAH. Regarding hemodynamics, we observed that patients with PoPH had higher COs and CIs than patients with I/H-PAH, which is similar to the findings of studies from western countries.

Previous reports have suggested that patients with PoPH have higher COs and CIs, lower PVRs, and higher exercise tolerances than those with IPAH [5, 22]. Similarly, in Study 1 (our study based on data from the nationwide registration system), patients with PoPH showed significantly higher COs, CIs, and higher exercise tolerances than that of patients with I/H-PAH, according to the registration forms from 2012 to 2013 (Table 1). Similarly, in Study 2 (our study at Chiba University Hospital), the patients with PoPH showed higher COs and CIs than the patients with I/H-PAH (Table 3). In patients with PoPH, higher COs associated with hyper-dynamic circulation may first occur due to overload and fluid retention, and may finally COs could reduce as PVR is raised [5, 19, 23]. Krowka et al. suggested a poor correlation between COs and most pulmonary hemodynamic parameters [19]. PAH-targeted therapy may increase CO in patients with high COs, resulting in a worsening of volume overload. The prognosis of these patients with high CO may be better even untreated, but it is still unclear.

Regarding sex, Kawut et al. showed that the females have a higher risk of PoPH [8]. The REVEAL registry has reported that 52% of patients with PoPH were females, and 79% of patients with IPAH were females (p < 0.001). The proportion of females in the IPAH group was significantly higher than in the PoPH group [11]. In Study 1 (our study based on data from the nationwide registration system), the registration forms showed a female-to-male ratio of 56% for PoPH, and of 57% for I/H-PAH (*p* = 0.84). The updated forms showed a female-to-male ratio of 57% for PoPH and of 67% for I/H-PAH (*p* = 0.14). In Study 2 (our study at Chiba University Hospital), the female ratio was 73% in PoPH and 72% in I/H-PAH (*p* = 0.95). Our studies showed a female-dominant trend in both groups, but the proportion of females was not different between the PoPH and I/H-PAH groups, unlike the data described in the REVEAL study.

Regarding the etiology of PoPH, Kawut et al. showed that autoimmune hepatitis was associated with a higher risk of PoPH and HCV was associated with a decreased risk of PoPH in patients evaluated for liver transplantation or pulmonary hypertension [8]. Conversely, a report from China showed that ≥ 50% patients with PoPH included in their study had hepatitis B virus infections [24]. In our Study 2 (study at Chiba University Hospital), the number of patients with viral hepatitis was greater than of those with autoimmune hepatitis, similar to the report from China. The etiology of patients with PoPH may have been affected by several backgrounds, including endemic diseases. Treatment may vary depending on the etiology.

Regarding treatment, the REVEAL study showed less PAH-targeted therapy in patients with PoPH than in those with IPAH. At enrollment, the ratio of patients without PAH-targeted therapy was 6% in patients with I/H-PAH, whereas it was 16% in patients with PoPH [11]. Considering monotherapy studies, Krowka et al. suggested that monotherapy with sildenafil had shown initial improvements in PVR at 3 months in patients with PoPH [25, 26]. Concerning the endothelin pathway, there was a report that showed blood concentration of endothelin-1 is increasing in patients with PoPH [27], so ERA is expected to be effective. In the PORTICO study, the first randomized study on PoPH, patients with PoPH treated with macitentan showed better PVR improvement than those treated with a placebo [15, 28]. On the other hand, the side effects associated with PAH-targeted therapy may sometimes differ between patients with IPAH and patients with PoPH. For example, some studies reported that progressive splenomegaly was developed in patients with PoPH as a complication of epoprostenol therapy [29, 30]. In patients with PoPH, clearance of PAH-targeted therapy could decrease with higher serum concentration, and may induce more side effects [28]. Patients with PoPH must be treated carefully. Regarding combination therapy, the evidence is limited to some case reports. Combination therapy (recommended for IPAH) was not recommended for PoPH in the 2015 European Society of Cardiology/European Respiratory Society guideline [31] or in the 6th world symposium on Pulmonary Hypertension [17] because most randomized studies on PAH therapy excluded patients with PoPH, and the efficacy/safety ratio of initial combination therapy for patients with PoPH has not been established.

Regarding our study, in Study 1 (our study based on data from the nationwide registration system), the results using the registration forms showed no significant differences in the number of patients receiving PAH-targeted therapy between patients with PoPH and those with I/H-PAH. The low proportion of patients with PAH being treated with combination therapy (11% vs. 20%) indicates that patients were not treated or were using only one drug at the time of registration because patients were often registered immediately after diagnosis (Table 1). However, using the updated forms, both groups showed increased proportion of patients with combination therapy. We detected a lower proportion of patients with PoPH receiving combination therapy compared with I/H-PAH patients (39% vs. 54%; *p* = 0.0524), but no significant differences in the proportion of patients receiving modern PAH therapy (including monotherapy) compared to patients with I/H-PAH (89% vs. 84%; *p* = 0.3535). The low number of patients receiving combination therapies may be due to the guidelines recommending monotherapy for PoPH treatment in the beginning and due to the patients being carefully monitored, while monitoring their liver function. Patients with PoPH were more likely to receive PDE5is than patients with I/H-PAH, but significantly less likely to receive ERAs and PGI_2_. The reason why PDE5i was often used was likely to be its ease of dosage adjustment. Regarding ERAs, bosentan is contraindicated for patients with moderate or severe liver damage and ambrisentan is contraindicated for patients with severe liver damage (it often causes edema as a side effect). This made it difficult to use ERAs in PoPH patients and seemed to result in their low usage. However, the data in this registry were obtained before macitentan became available, and we expect the use of ERAs to increase in the future. (Table 2). Similarly, in Study 2 (our study at Chiba University Hospital), we found similar proportions of patients receiving modern PAH therapy (64% vs. 85%; *p* = 0.1442), although fewer patients with PoPH were treated with combination therapy (27% vs. 67%; *p* = 0.0191) (Table 4). Although their liver function was less severe (5 of 6 patients with cirrhosis had Child–Pugh A), 5 of 11 had to discontinue medication owing to its adverse effects (Table 5). Actually, PAH-targeted therapy may be useful for patients with PoPH. However, the medications had to be carefully chosen because PAH-targeted drugs are mainly metabolized in the liver. We consider that patients with PoPH should be initiated on monotherapy carefully at the beginning of the treatment.

In Study 1 (our study based on data from the nationwide registration system), both the I/H-PAH and PoPH groups showed significant improvements in trans-tricuspid pressure gradient between the registration and updated forms (I/H-PAH; *p* < 0.0001, PoPH; *p* = 0.0004) despite a low proportion of patients with PoPH receiving combination therapy. In Study 2 (our study at Chiba University Hospital), hemodynamic changes from baseline to the last follow-ups in patients with PoPH showed a tendency to improve, albeit not significantly (Fig. 1). The small sample size may have affected this result; only five patients with PoPH underwent follow-up RHCs.

Regarding survival, previous studies have demonstrated that the survival of patients with PoPH is worse than that of patients with IPAH [11–13]. A report from China showed that 57% of 14 patients with PoPH died during a 26-month follow-up period (no patients received vasodilators) [24]. However, Pavec et al. reported that the survival of patients with PoPH was similar to that of patients with IPAH, and 51% patients had Child–Pugh A scores [32]. As Study 1 (our study based on data from the nationwide registration system) did not contain data on survival, we analyzed survival in Study 2 (our study at Chiba University Hospital), which showed a similar demographic to that of Study 1.

In Study 2, only 6 of the 11 patients had cirrhosis, and 5 of 6 had Child–Pugh A scores (less severe liver disease). Figure 2 shows similar overall and disease-specific survival between PoPH and I/H-PAH groups, even though patients with PoPH tend to receive monotherapy. Considering the similar survivals between the two groups and higher ratio of adverse events in patients with PoPH, introducing treatment with monotherapy appears to be better option in patients with PoPH.

There were some limitations to this study. Study 2 was a retrospective, single-center study, with a small sample size. Figure 1 shows the hemodynamic changes from baseline to the last follow-up in Study 2. However, a small sample size may have influenced these results. Study 1 did not contain data on survival (hence, survival was analyzed in Study 2). Clinical characteristics of healthy volunteers were not included; thus, a standard control was absent from both studies.

In conclusion, Japanese patients with PoPH showed higher COs and CIs, and better exercise tolerances than patients with I/H-PAH, as reported in western countries. Further studies are needed to clarify whether Japanese patients with PoPH should be carefully treated with monotherapy as an initiation therapy.

## Data Availability

The study database was anonymized, and the study complied with the requirements of the Japanese Ministry of Health, Labour and Welfare. The datasets analyzed during the current study are not publicly available, but are available from the corresponding author on a reasonable request and with permission of our department.

## Abbreviations

BNP: Brain natriuretic peptide
CI: Cardiac index
CO: Cardiac output
ERA: Endothelin-receptor antagonist
FEV_1_: Forced expiratory volume in 1 s
HCV: Hepatitis C virus
I/H-PAH: Idiopathic/heritable PA
mPAP: Mean pulmonary artery pressure
NYHA: New York Heart Association
PAWP: Pulmonary artery wedge pressure
PAH: Pulmonary arterial hypertension
PDE5i: Phosphodiesterase type 5 inhibitor
PGI_2_: Prostaglandin I_2_
PoPH: Portopulmonary hypertension
PVR: Pulmonary vascular resistance
RHC: Right heart catheterization
6MWD: 6 minute walk distance
